# Role of CT and MRI in Cardiac Emergencies

**DOI:** 10.3390/tomography8030112

**Published:** 2022-05-23

**Authors:** Carlo Liguori, Stefania Tamburrini, Giovanni Ferrandino, Silvio Leboffe, Nicola Rosano, Ines Marano

**Affiliations:** Department of Radiology, Ospedale del Mare-ASL NA1 Centro, Via Enrico Russo 11, 80147 Naples, Italy; gianni.ferrandino90@gmail.com (G.F.); silvio.leboffe@gmail.com (S.L.); nicola.rosano89@gmail.com (N.R.); maranoines@gmail.com (I.M.)

**Keywords:** cardiac radiology, cardiac-CT, cardiac MRI, CT angiography, acute coronary syndrome, myocarditis, cardiomyopathy

## Abstract

Current strategies for the evaluation of patients with chest pain have significantly changed thanks to the implemented potentiality of CT and MRI. The possible fatal consequences and high malpractice costs of missed acute coronary syndromes lead to unnecessary hospital admissions every year. CT provides consistent diagnostic support, mainly in suspected coronary disease in patients with a low or intermediate pre-test risk. Moreover, it can gain information in the case of cardiac involvement in pulmonary vascular obstructive disease. MRI, on the other hand, has a leading role in the condition of myocardial damage irrespective of the underlying inflammatory or stress related etiology. This article discusses how radiology techniques (CT and MRI) can impact the diagnostic workflow of the most common cardiac and vascular pathologies that are responsible for non-traumatic chest pain admissions to the Emergency Department.

## 1. Introduction

Most patients with cardiovascular emergencies present with chest pain and/or dyspnea. In this scenario, cardiac and non-cardiac etiologies must be taken into consideration [1]. Cardiac causes include ischemic and non-ischemic pathologies: acute coronary syndrome (ACS), stress-induced cardiomyopathy, and myo/pericarditis. Chest pain related to non-cardiac pathologies is mainly represented by aortic dissection, esophageal alterations, pulmonary embolism, inflammatory lung disease, and costochondritis [2]. The question of whether a patient presenting with acute chest pain may be safely discharged from the emergency department (ED) remains one of the most common and contentious clinical conundrums facing physicians [3]. Distinguishing visceral from musculoskeletal pain is the first essential step in the diagnostic approach. Most nontraumatic musculoskeletal chest wall pain (42%) is attributed to costochondritis which is diagnosed by physical examination [4]. Contrastingly, once visceral pain is suspected a stepwise diagnostic algorithm must be followed in order to reach a final diagnosis. This strategy usually includes a clinical evaluation (medical history and physical examination); laboratory samples (mainly cardiac troponins, B-type natriuretic peptide [BNP], N-terminal proBNP [NT-proBNP], D-dimer); and imaging methods (electrocardiogram [ECG], chest radiograph [CXR], transthoracic echocardiography [TTE], computed tomography [CT]). In a subacute phase, Magnetic Resonance Imaging (MRI) should be utilized [1,5,6,7,8,9,10,11,12,13,14]. In the context of current guidelines and mostly applied diagnostic protocols, the aim of this article is to highlight the pivotal role played by diagnostic radiological exams (CT and MRI) in the management of patients who present in the emergency setting with a cardiac pathology. 

## 2. Myocarditis 

Myocarditis, a non-ischemic inflammatory disease of the myocardium is a cause of myocardial injury. It may be present in acute, fulminant, subacute and chronic forms [15]. Injury can manifest itself across a wide spectrum of clinical severity: from subclinical disease to myocarditis with preserved cardiac function, to more severe cases that result in reduced systolic or diastolic function, arrhythmia, and rarely, hemodynamic collapse and cardiogenic shock [16]. According to postmortem analysis, myocarditis accounts for up to 12% of sudden cardiac deaths in young adults [16,17]. Acute myocarditis is defined as a period of <1 month between the onset of symptoms and diagnosis [16,18,19] and it must be differentiated from acute coronary syndromes and acute pericarditis given the overlapping clinical features [15]. In a recent metanalysis, Tronvall et al. reported that approximately one-third of those patients presenting with acute coronary syndrome without substantial coronary artery disease are ultimately diagnosed with acute myocarditis [20]. Myocarditis is driven by an immune response directed at cardiomyocytes. The initial trigger in developed countries is viral infection [21]. Noninfectious causes of myocardial inflammation include autoimmune and immune-mediated disease. Several drugs and medications have also been reported as a cause of myocarditis [22]. Although the diagnostic gold standard is still considered the histologic evaluation of the myocardium showing focal or diffuse inflammatory cellular infiltration [23], nowadays endomyocardial biopsy is rarely performed [16]. In the emergency setting diagnostic criteria is mainly focused on electrocardiographic parameters and elevated troponin levels and a functional and structural evaluation using cardiac imaging (mainly cardiac MRI (CMR) which is considered mandatory) [24]. 

CMR can identify three diagnostic features during an inflammatory myocardial process: edema, hyperemia, and necrosis or fibrosis [25].

These three targets have been proposed for the diagnosis of acute myocarditis since the first consensus on Cardiovascular Magnetic Resonance (CMR) in Myocardial Inflammation in 2009—the “Lake Louise Criteria” (LLC)—and were subsequently revised and implemented in 2018 [26]. Traditional T2-weighted imaging and T2 mapping techniques can assess the presence of edema as a physiological response triggered by damage to living tissues.

Hyperemia is the first stage of the inflammatory response, a process characterized by a change in blood flow in the damaged area. T1w images after the administration of a gadolinium-based contrast agent show an early and intense gadolinium enhancement compared with a noninflamed myocardium. 

Necrosis or fibrosis are both consequences of prolonged or severe tissue damage. This mechanism contributes to expanding the interstitium, increasing the volume of contrast media in the extracellular space. Necrosis and fibrosis might be evaluated using late gadolinium enhancement (LGE) imaging. Further, native T1 mapping is considered a useful tool for the evaluation of fibrosis. 

Furthermore, extracellular volume (ECV) is also considered an indicator of extracellular space expansion which typically occurs in fibrotic changes and can be assessed using T1 maps acquired pre- and post-administration of contrast media. It has a complementary role to LGE due to its ability to detect milder and diffuse fibrosis. The MyoRacer trial [27] found similar incidence of LGE in patients with acute and chronic myocarditis; however, the latter had relatively higher intramural enhancement and a significantly lower incidence of lateral wall involvement. 

During the SARS-CoV2 pandemic, several cases of myocarditis were observed. 

Similar to other viral pathogens, in this setting myocardial impairment was the result of direct viral cell damage and an autoimmune reaction. Consequently, CMR findings do not differ from what was already described in the Lake Louise criteria [25]. 

**Figure 1:**
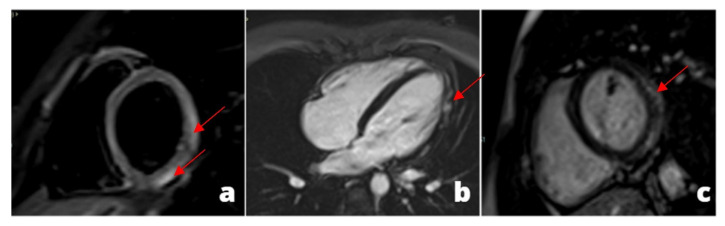
A young adult presenting at emergency department with acute chest pain, subtle ST alterations in the lateral wall of the left ventricle and cardiac enzymes elevation. Cardiac MRI was performed 48 h later demonstrating edema in the inferior-lateral wall of the left ventricle (**a**) associated with hyperemia in early gadolinium enhancement (**b**) and post-inflammatory fibrosis using late gadolinium enhancement sequences (**c**).

As the pharmacokinetics of iodinated and gadolinium- based contrast agents are similar, the technique of late myocardial enhancement could also be applied on MDCT scans [28]. Decreased tube voltage (70–80 kVp) and increased contrast agent volume may strengthen damaged myocardial density and enhance scar or fibrosis visualization in delayed phase cardiac MDCT even with lower signal-to-noise ratio. Furthermore, multi-energy scanners may provide additional improvement to the potential of MDCT. It has been demonstrated that monochromatic images with optimal energy levels derived by multi-energetic acquisition yield a better contrast-to-noise ratio than conventional single-energy polychromatic images that are commonly used for late enhancement [29]. With the optimized late enhancement technique, MDCT could be a promising “one stop shop” exam and an alternative to CMR, especially in patients with an infarct-like presentation of acute myocarditis (Figure 1).

## 3. Pericarditis

Pericardial inflammation may occur in quite a varied spectrum of conditions, including infections (viral, bacterial, fungal and tubercular); autoimmune diseases (such as LES, scleroderma, rheumatoid arthritis); primary or secondary pericardial tumors; and chronic kidney disease. Furthermore, pericarditis may be triggered by direct pericardial injury (surgery, mediastinal radiotherapy) and cardiac damage (transmural infarction and Dressler syndrome). Up to 30% of cases have no defined cause and are consequently classified as idiopathic [30].

**Figure 2:**
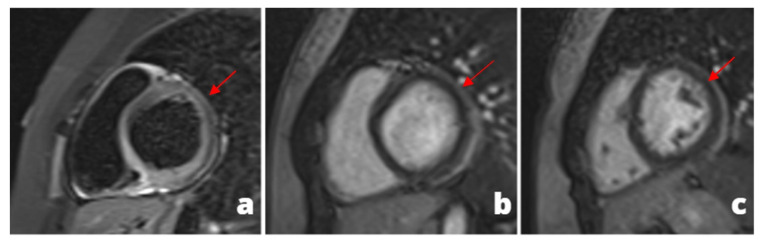
A 28 y/o man presented at the emergency department with fever and chest pain. At the admission, cardiac enzymes were elevated and ECG did not show any alteration. Cardiac MRI was performed 1 day later and revealed pericardial alteration: presence of edema (**a**) and post-inflammatory fibrosis (**b**,**c**).

CT and MRI can equally demonstrate pericardial effusion and pericardial thickening. A 4 mm thickness is conventionally indicated as the upper limit of normal, although it must be emphasized that pericarditis may be present even when the pericardium is within normal limits. Stranding of the pericardiac fat tissue can be an ancillary finding [31]. MRI better depicts pericardial enhancement (on spin echo T1 and LGE sequences) and myocardial enhancement, and the combination of the two (myopericarditis) entails a higher risk of complications [32] (Figure 2). 

In some cases, acute inflammation causes permanent fibrotic changes and calcium deposition in the pericardium, resulting in constrictive pericarditis. In this condition, the stiffening of the pericardium affects ventricular filling. During inspiration RV filling prevails whereas during expiration LV filling is enhanced. The combination of morphologic changes in the pericardium, the remodeling (tubing) of ventricular cavities and biatrial enlargement are indirect signs of constrictive pericarditis. MRI, however, permits a direct demonstration of functional alterations: real-time cine sequences acquired during free-breathing show flattening of the interventricular septum at inspiration, followed by a return to normal convexity at the end of expiration (septal bounce). 

## 4. MINOCA: Myocardial Infarction with Non-Obstructive Coronary Arteries

MINOCA occurs in 1–15% of acute MI cases in patients presenting with acute ST-segment elevation MI or non-ST segment elevation MI [33,34]. The incidence of major cardiovascular (CV) events (MACE) in MINOCA has increased in the past few years [35]. Patients with MINOCA are often younger, more likely to be women, and less likely to have dyslipidemia [36,37]. Approximately one-third of patients have been reported to present suspected STEMI within an emergency setting and the remaining majority as NSTEMI patients undergoing subsequent angiography [38,39]. The diagnosis of MINOCA is made according to the Fourth Universal Definition and must meet three criteria: first, a definitive diagnosis of MI; second, the absence of culprit obstructive coronary artery disease (epicardial coronary artery stenosis ≥ 50%) in an infarct-related epicardial artery during coronary angiography following acute MI; third, there is no overt systemic etiology for the presentation (e.g., myocarditis and pulmonary embolism). MINOCA disorders can be classified within the Fourth Universal Definition of MI. They may meet criteria for type 1 MI where epicardial coronary artery disorders are diagnosed, or type 2 MI due to endothelial dysfunction or oxygen supply and demand mismatch, or myocardial injury [40]. When a diagnosis of MINOCA is made, an invasive and adjunctive investigation should be considered with the appropriate utilization of intravascular imaging, coronary function testing and subsequent imaging to assess myocardial disorders without coronary involvement [41]. Because plaque rupture, erosion, ulceration, calcific nodules and intraplaque hemorrhage are common in MINOCA, coronary intravascular ultrasound (IVUS) or optical coherence tomography (OCT) are useful in assessing ‘missed’ obstructive disease or dissection [42].

CMR should be performed as soon as feasible after MINOCA is identified and it is therefore recommended within 7 days of presentation because delayed imaging can sometimes result in some features no longer being evident [26,33]. CMR can identify myocardial activity, inflammation, tissue morphology, myocardial oedema and scarring. Late gadolinium enhancement, if present localizes the site of myocardial damage and the pattern of distribution can suggest the diagnosis: subendocardial or transmural enhancement is typical of an ischaemic aetiology, whereas the absence of late gadolinium enhancement may be in keeping with microvascular dysfunction or a non-cardiac cause [43]. 

**Figure 3:**
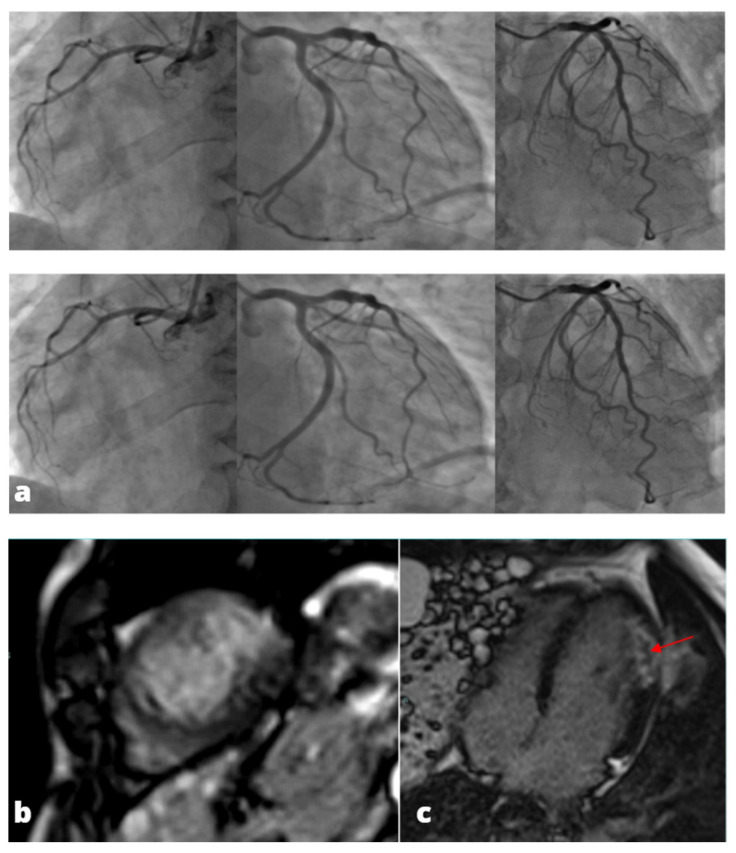

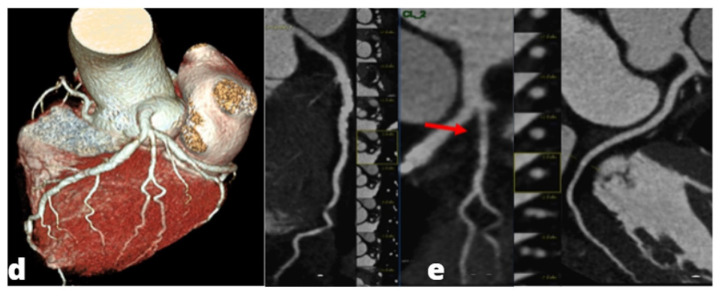
MINOCA-Miocardial Infarct non obstructive coronaries. The patient presented to the emergency department with acute chest pain, ECG and cardiac enzymes suggestive of STEMI in the lateral wall of the left ventricle. The patient was readily referred to coronary angiography and cardiac catheterization showed absence of obstructive stenoses in the coronary tree (**a**). One day later a cardiac MRI was performed demonstrating a subacute ischemic scar in the lateral wall of the medium-apical left ventricle ((**b**)-LGE short axis view, (**c**)-LGE four chambers view). In the context of transmural late gadolinium enhancement, a constantly hypointense central area is present, representing a microvascular obstruction zone (red arrow). A cardiac CT was subsequently performed in order to assess coronary wall features and the presence of atherosclerosis. CT images in volume rendering (**d**) and multiplanar oblique reconstruction (**e**) demonstrated a non-calcified atherosclerotic alteration in an obtuse marginal vessel, serving the infarcted zone. A partly calcified atherosclerotic pathology was present in the anterior descending artery.

CT coronary angiography (CCTA) is not indicated in these patients [9,10] due to the presence of ECG alterations and enzyme elevation that were recorded, but it may be of value when diagnostic uncertainty remains after intravascular imaging. In this group of patients, CT can assess the presence of intramural hematoma, dissection and acute coronary plaque disease. A negative CT study is associated with excellent prognosis and it allows the remaining work-up of non-ischemic causes to be redirected. Moreover, a normal CT study also has important management implications because the use of antiplatelet and statin therapy can be considered pointless when the atherosclerotic wall changes are absent. However, it is important to note that CCTA may be less accurate in an acute setting because of general illness. Therefore, the prognostic implications of a negative CCTA may not apply to MINOCA patients (Figure 3).

## 5. Takotsubo Cardiomyopathy

Takotsubo cardiomyopathy (TTC), also known as stress cardiomyopathy is an acute transient left ventricle dysfunction in which there is a sudden and reversible myocardial stunning in the absence of occlusive coronary artery disease [43]. TTC is usually triggered by physical or emotional stress in one-third of the cases. Clinically, it mimics acute myocardial infarction and patients typically refer chest pain and/or dyspnea. It is responsible for about 1% to 2% of patients presenting with acute myocardial injury and it often presents as a MINOCA “mimic” [43]. TTC predominantly affects postmenopausal women (90%) and accounts for approximately 5–6% of female patients presenting with suspected ST-elevation myocardial infarction (STEMI). Therefore, cardiac imaging tools have a crucial role in these patients. Coronary angiography is necessary to exclude pathological coronary stenosis and CCTA should be considered as an alternative diagnostic tool in the case of low or medium pre-test risk patients because of its high negative predictive value. On the other hand, transthoracic echocardiography is the first non-invasive imaging modality for an early evaluation of left ventricle systolic and diastolic function [44]. 

CMR is indicated in all patients presenting with suspected Takotsubo. CMR allows for the multiparametric quantification of myocardial tissue characteristics such as the absence of late gadolinium enhancement (typically) and of late enhancement on delayed contrast sequences. These findings differentiate Takotsubo cardiomyopathy from anterior STEMI in which necrosis in the wall is present in most patients even when there is no-reflow or microvascular obstruction [45,46]. CMR detects myocardial edema on T2-STIR that directly relates to the water content in the myocardial wall. In Takotsubo cardiomyopathy, edema is typically located in the apical mid-ventricular planes, sparing the basal plane, and matches the wall-motion abnormalities seen on cine MRI [45,46,47,48].

**Figure 4:**
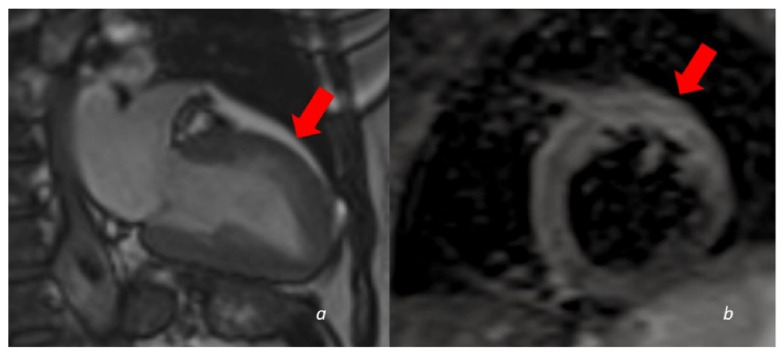
A 60 y/o woman presenting with acute chest pain and Troponin elevation with ECG alterations in the anterior leads. Echocardiogram showed dyskinesia of the anterior middle wall of the left ventricle. Subsequent cardiac MRI (24 h later) demonstrated dyskinetic movement in the anterior wall of the left ventricle ((**a**)-cine images frame in the systolic phase showing anterior bulging), associated to edema in the anterior wall (**b**). No LGE was found in the myocardial wall.

Less frequent can be the reverse presentation (reverse Takotsubo syndrome) where the basal left myocardium can be affected (Figure 4).

## 6. Acute Coronary Syndrome

Acute coronary syndrome (ACS) refers to a group of diseases in which blood flow to the heart is decreased. It is a manifestation of CHD (coronary heart disease) which usually results in plaque disruption in the coronary arteries (atherosclerosis) and the formation of thrombus. In some cases, ACS may be due to vasospasm with or without underlying atherosclerosis, but in both cases it determines a reduction in blood flow to a part of the heart, resulting first in ischemia and then infarction.

ACS includes several conditions: ST-segment elevation myocardial infarction (STEMI), non-ST-segment elevation myocardial infarction (NSTEMI), and unstable angina, and it is responsible for one third of total deaths in people older than 35 [49]. 

In patients with suspected ACS, it will first be necessary to classify the patient in one of the two major sub-categories (STEMI and NSTEMI) and subsequently to distinguish the N-STEMI subtype (N-STEMI and unstable angina). STEMI is representative of an acute myocardial infarction with evidence of myocardial necrosis. Most of the patients presenting with ischemic symptoms and persistent ST-segment elevation on the electrocardiogram (ECG) > 20 min will show a typical rise in biomarkers of myocardial necrosis and progress to Q-wave myocardial infarction [50]. Patients with acute chest pain but no persistent ST-segment elevation may have a variety of ECG abnormalities such as transient ST-segment elevation, persistent or transient ST-segment depression, inversion of the T wave, flat T waves, or pseudo-normalization of the T waves up to a normal ECG. Cardiomyocyte necrosis [myocardial infarction without ST-segment elevation (NSTEMI)] or, less frequently, myocardial ischemia without cell damage (unstable angina) may be observed in the myocardium [10]. 

### Suspected Acute Coronary Syndrome 

**Figure 5:**
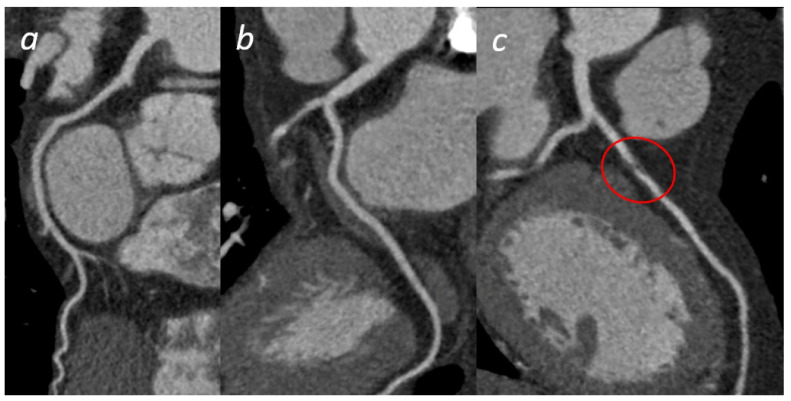

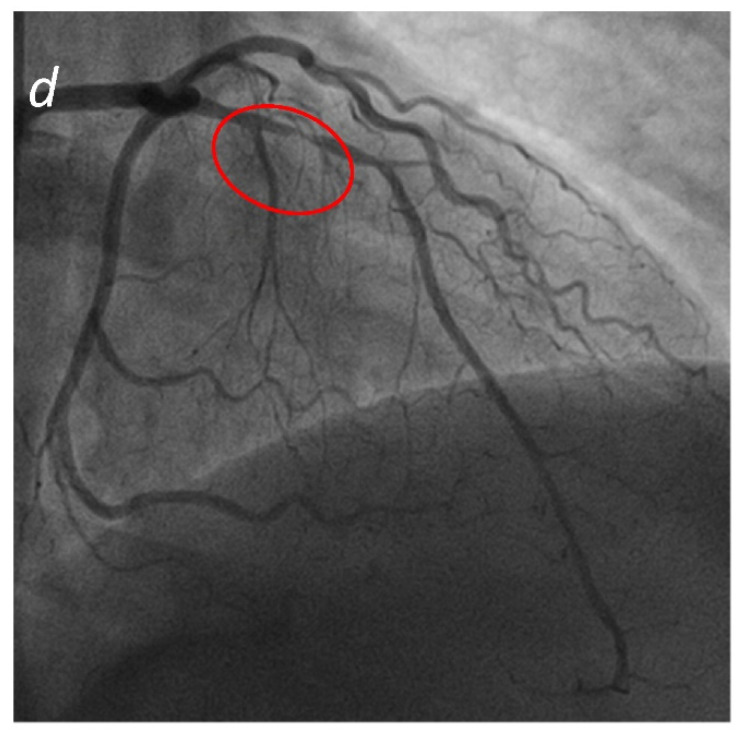
Acute Coronary Syndrome. A patient presenting to the emergency department with acute chest pain. No significant cardiac enzyme elevation and ECG alteration. Cardiac CTA shows absence of significant stenoses in the right coronary artery (**a**) and the left circumflex (**b**); a significant stenosis in the proximal anterior descending artery (**c**) is present, confirmed by subsequent coronary angiogram (**d**).

In patients with a high risk of ACS, a prompt invasive coronary angiography and/or revascularization should be performed to salvage the viable myocardium. In such a scenario, radiology techniques (CT or MRI) should not be employed, they should only be used in the post-treatment phase for assessing the extent of myocardial damage. In contrast, in patients with a low to moderate risk of ACS alongside the non-invasive clinical instrumental standard evaluation, radiological techniques and in particular CT can play a key role in making a faster diagnosis, compared to standard of care [51,52,53]. Standard protocol is associated with a long stay in the emergency room or coronary care unit waiting for any changes in the ECG or troponin to occur or waiting for stress tests, the results of which are emergency room overcrowding and an increase in cost. State-of-the-art CT scans (≥64 slices) can acquire images in moments, thereby reducing the possibility of artifacts and allowing the quick and reliable exclusion of ACS without ST-segment elevation by displaying the main coronary vessels and their main branches. The ROMICAT II and ACRIN studies have shown that CTA can lead to rapid and safe discharge from ED (47% vs. 12%, *p* < 0.001 and 49.6% vs. 22.7%, *p* < 0.001, respectively), reducing hospitalization times (8.6 h vs. 26.7 h, *p* < 0.001 and 18.0 h vs. 24.8 h, *p* < 0.001, respectively) and showing a better cost–benefit ratio than standard-of-care, thanks to the high negative predictive value of the CTA (excluding coronary stenosis > 50%) [54,55]. The main advantage of coronary CT is the possibility to evaluate the vessel wall with consequent plaque characterization. CT can also exclude or detect any serious complications of acute myocardial infarction (pulmonary edema and acute mitral regurgitation, due to the ischemic involvement of the papillary muscles, ventricular septal defect, free wall rupture, etc.) [56,57,58]. A type 1 acute myocardial infarction (AMI) is triggered by fissuring and the subsequent rupture of the vulnerable plaque, resulting in obstructive coronary stenosis or occlusion. Thus, a culprit lesion is often characterized at CT evaluation by a ≥70% stenosis with mixed or mainly noncalcified plaque [59]. A Type 2 AMI is caused by a mismatch of blood supply and demand. Type 2 AMI is often referred to as myocardial infarction with nonobstructive coronary arteries (MINOCA). In this context, cardiac-CT allows the evaluation of the presence of non-obstructive plaques (<50%) with vulnerable characteristics, as well as the presence of myocardial damage in the possible distribution area of the suspected lesion [52] (thanks to the acquisition of LIE-Late Iodine Enhancement). There are still major limitations related to available instruments, radiation dosage, and low positive predictive value in prognostic terms, especially in patients with calcifications, obesity and suboptimal heart rate. Coronary CT can identify coronary stenosis that is not functionally significant and decrease the number of coronary angiography procedures not followed by revascularization [60]. Theoretically, cardiac MRI is the ideal method for studying ACS. It allows not only the evaluation of myocardial perfusion and parietal motility abnormalities but the distinction between scar tissue and recent infarction. CMR, combining examination at rest and with stress can be used in acute chest pain that is suspected ACS. In clinical practice, in the emergency setting it carries very little application in the diagnostic phase [61] (Figure 5).

According to recent literature the integration of derived fractional flow reserve in CT (FFR-CT) studies using computational fluid dynamics gives the chance to enhance CT diagnostic accuracy. Moreover, in the emergency setting the use of FFR-CT allows a safer patient management thanks to the opportunity to detect, in the case of negative study, cardiac revascularization in chest pain patients. At the same time, it allows a significant reduction in medical cost [62].

Alternatively, it is now possible to combine a coronary CT angiography with myocardial CT perfusion to define the hemodynamic significance of a coronary stenosis. Combined stress CT perfusion and CTA are strictly correlated to a shortened period of hospitalization and lower costs [63].

## 7. Pulmonary Embolism and Cardiac Involvement

Pulmonary Embolism (PE) is defined as the embolic occlusion of the pulmonary arterial system. In 95% of cases, it is caused by detached clots from deep vein thrombosis (DVT) of the lower extremities.

The persistence or the recurrence of pulmonary embolic events is defined as Chronic Pulmonary Thromboembolism (CTEP) which can lead to delayed pulmonary hypertension (PH), a potentially fatal event [64,65,66].

The overall mortality of acute PE is 10–30%, making it the third most common cause of cardiovascular death and accounting for 300,000–370,000 deaths in Europe every year [67,68].

The wide spectrum of symptoms present in acute PE poses a diagnostic problem. It ranges from asymptomatic emboli with nonspecific clinical symptoms to massive life-threatening embolisms usually associated with right heart failure. 

The most common symptoms comprise chest pain, dyspnea, cough, hemoptysis and syncope but massive PTE can also lead to acute shock-inducing tachycardia, hypotension, tachy-/orthopnea, hypoxemia, hypocapnia, acute Right Ventricular Dysfunction (RVD) and even sudden death [68,69]. 

RVD indicates a potential emergency because it represents a rapidly progressive congestion syndrome resulting from impaired filling and/or reduced flow out of the right ventricle. It is triggered by acute pressure overload and is associated with a poor prognosis and a high risk of early mortality within 30 days [70].

D-dimer is the most sensitive but non-specific diagnostic test for acute PE. Specificity improvements can be made using a cut-off value of 1000 ng/mL in patients with a low pre-test clinical probability score and of 500 ng/mL in patients with a moderate clinical probability score. Lower values safely exclude pulmonary embolism and unnecessary further diagnostic investigations [71]. The role of CT pulmonary angiography (CTPA) in the diagnosis of acute PTE is widely validated in the literature [72].

To assess the severity of PE, the commonly used CT Obstruction Index (CTOI) described by Qanadli et al. [73] calculates the sum of the individual scores per artery, divided by 40 (the maximum total score) and converted to a percentage. The score is calculated by assigning a unit value to each pulmonary artery segment with thromboembolism (max 10 for each lung) and multiplying it by a weight factor (1 for partial obstruction or 2 for total obstruction). As suggested by the authors, two groups of patients are identified, the high-risk sub-group with a CTOI ≥ 20/40 (50%) and the low-risk sub-group with a CTOI < 20/40 [73].

The role of the Qanadli index in immediate risk stratification is validated but no correlation was observed between the obstruction index and prognosis [74,75,76,77,78,79,80,81,82,83,84,85,86].

Rotzinger et al. recently reported that patients with acute PE, excluding those with comorbid cardiopulmonary or pulmonary neoplasms and with a CTOI greater than 40% had significantly higher mortality than those with a CTOI less than 20% [75].

Complications of acute PTE include right ventricular dysfunction and pulmonary infarction. Signs of right heart strain on CTPA are an increased RV/LV diameter ratio > 1 measured on the short-axis or four chambers plane, an enlargement of the pulmonary trunk > 29 mm, flattening or inverse bowing of the interventricular septum and the reflux of contrast material into the inferior vena cava and hepatic veins [76,77,78,87]. Several studies have validated their role as a predictor of short-term mortality (within 30 days) and adverse clinical events in patients with acute PE.

Ayöz et al. [78] described higher troponin levels in patients with RV/LV > 1.

**Figure 6:**
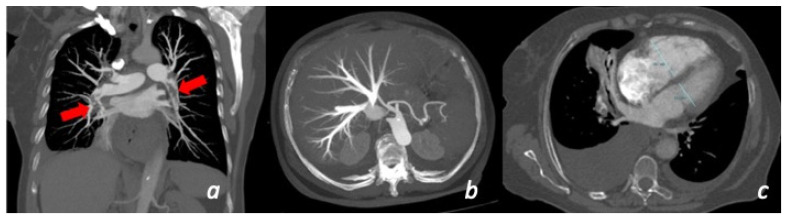
Patient with chest pain and dyspnea for more than 10 days with recent worsening of symptoms. CT shows the presence of massive bilateral pulmonary thrombo-embolism (red arrows) in the right and left lower lobe pulmonary artery (**a**). Secondary overload of the right-side circulation: enlargement of the right ventricle (**c**) and secondary tricuspid insufficiency (**b**).

Recently, further studies have focused attention on the additional parameters of RVD; Cozzi et al. showed that acute coronary sinus dilatation (>9 mm) increased the risk of all-cause death within 30 days [21]. Langroudi et al. [80] investigated the association between CTOI and atrial size in patients with acute PE, showing that a higher clot load is associated with a smaller LA size and increased RA/LA ratios [80]. According to Aviram et al. [81], a decreased volume of LA (<62 mL) is the best predictor of adverse outcome. MRI should be considered if radiation is a concern, particularly in pregnant or young patients, as well as in patients with contraindications to iodinated contrast, mainly prior severe allergic reaction and severe renal insufficiency. It can even be helpful without the use of intravenous contrast material at the cost of lower sensitivity for segmental and subsegmental emboli [88]. In the same group of patients, lung scintigraphy, i.e., ventilation (V) and perfusion (Q) scans can also serve as an alternative to CTPA given the significantly lower radiation dose and the use of non-iodinated agents. The sensitivity of VQ scanning can be improved using three-dimensional VQ single-photon emission computed tomography (SPECT) or hybrid SPECT/CT, achieving diagnostic accuracy that is similar to CTPA [89]. In patients with relative contraindications to iodinated contrast, e.g., prior to mild allergic reaction or mild renal insufficiency, CTPA is still the modality of choice after respective preventive measures, particularly for patients who are intolerant to flat positioning (Figure 6).

## 8. Conclusions

The use of radiology techniques, CT, and MRI in the context of cardiac emergencies has enormously increased thanks to the massive use of cardio-synchronized CT in the last few years.

CT in the emergency room allows for an expedited work-up of chest pain patients thanks to its very high (99%) negative predictive power for obstructive coronary artery disease. 

Appropriate CT utilization can lower healthcare costs and reduce radiation exposure. 

Nowadays, the gradual integration of FFR-CT or the use of pharmacological stress in the emergency settings may significantly improve the diagnostic performance in the near future.

Modern scanners allow a real time evaluation, in addition to coronary circulation and assessment of the pulmonary and aortic vascular systems, translating the myth of the “one stop shop investigation” of the patient with chest pain into a clinical emergency routine.

As it is intended for use within 3–5 days of symptom presentation and not in emergency conditions, MRI has recently become an irreplaceable tool in specific conditions once the presence of significant coronary obstructions has been excluded.

Cardiac MRI has a wide range of applications which allows patient risk stratification if performed soon after the onset of chest pain. It can identify the myocardial area at risk, quantify eventual microvascular obstruction and identify intramyocardial hemorrhage.

MRI can also give prognostic information thanks to the novel parametric mapping for tissue characterization. In some more complex emergency scenarios cardiac MRI helps to accurately classify those patients with atypical clinical features, such as MINOCA or Takotsubo cardiomyopathy cases. In doing so, different and adequate treatment can be determined.

Regarding CT, recent technological advances have made it possible to significantly reduce the exposure dose problem and the speed of execution. A technological implementation is desirable regarding the MRI side, especially for the improvement of acquisition times which are decisive in the emergency setting

Radiology has gained a central role in the diagnostic work-up of the patient with acute chest pain. Due to its potential, it has reached the chance to evaluate not only the vascular and coronary arteries but also to identify myocardial diseases associated with an emergency pain condition. It is therefore indispensable for the modern radiologist to acquire familiarity with diagnostic findings and the expression of acute cardiac damage, in order to potentially identify them in daily practice.

## Data Availability

Not applicable. Technical support or donations in kind (e.g., materials used for experiments) are also not applicable.

## References

[1] Chughtai A., Kazerooni E.A. (2007). CT and MRI of acute thoracic cardiovascular emergencies. Crit. Care Clin..

[2] Dong T., Parizher G., Jaber W.A. (2022). Triaging down the 2021 chest pain guidelines. JACC Case Rep..

[3] Bhatt D.L., Taqueti V.R. (2017). Out with the old rule-out: Raising the bar for acute chest pain evaluation with randomized trials of cardiac imaging. JACC Cardiovasc. Imaging.

[4] Grani C., Senn O., Bischof M., Cippa P.E., Hauffe T., Zimmerli L., Battegay E., Franzen D. (2015). Diagnostic performance of reproducible chest wall tenderness to rule out acute coronary syndrome in acute chest pain: A prospective diagnostic study. BMJ Open.

[5] Pope J.H., Aufderheide T.P., Ruthazer R., Woolard R.H., Feldman J.A., Beshansky J.R., Griffith J.L., Selker H.P. (2000). Missed diagnoses of acute cardiac ischemia in the emergency department. N. Engl. J. Med..

[6] Mueller C., Giannitsis E., Christ M., Ordonez-Llanos J., deFilippi C., McCord J., Body R., Panteghini M., Jernberg T., Plebani M. (2016). Multicenter evaluation of a 0-hour/1-hour algorithm in the diagnosis of myocardial infarction with high-sensitivity cardiac troponin T. Ann. Emerg. Med..

[7] Reichlin T., Lockwood S.J., Conrad M.J., Nof E., Michaud G.F., John R.M., Epstein L.M., Stevenson W.G., Jarolim P. (2016). Early release of high-sensitive cardiac troponin during complex catheter ablation for ventricular tachycardia and atrial fibrillation. J. Interv. Card Electrophysiol..

[8] Arslan M., Schaap J., Van Gorsel B., Budde R.P., Bekkers S.C., Van Cauteren Y.J., Damman P., Habets J., Dubois E.A., Dedic A. (2021). Coronary CT angiography for improved assessment of patients with acute chest pain and low-range positive high-sensitivity troponins: Study protocol for a prospective, observational, multicentre study (COURSE trial). BMJ Open.

[9] Collet J.P., Thiele H. (2020). The ‘Ten Commandments’ for the 2020 ESC Guidelines for the management of acute coronary syndromes in patients presenting without persistent ST-segment elevation. Eur. Heart J..

[10] Collet J.P., Thiele H., Barbato E., Barthelemy O., Bauersachs J., Bhatt D.L., Dendale P., Dorobantu M., Edvardsen T., Folliguet T. (2021). 2020 ESC Guidelines for the management of acute coronary syndromes in patients presenting without persistent ST-segment elevation. Rev. Esp. Cardiol..

[11] Mauro C., Vriz O., Romano L., Citro R., Russo V., Ranieri B., Alamro B., Aladmawi M., Granata R., Galzerano D. (2020). Imaging cardiovascular emergencies: Real world clinical cases. Heart Fail. Clin..

[12] Task Force M., Montalescot G., Sechtem U., Achenbach S., Andreotti F., Arden C., Budaj A., Bugiardini R., Crea F., Cuisset T. (2013). 2013 ESC Guidelines on the management of stable coronary artery disease: The task force on the management of stable coronary artery disease of the European society of cardiology. Eur. Heart J..

[13] Meijboom W.B., Meijs M.F., Schuijf J.D., Cramer M.J., Mollet N.R., van Mieghem C.A., Nieman K., van Werkhoven J.M., Pundziute G., Weustink A.C. (2008). Diagnostic accuracy of 64-slice computed tomography coronary angiography: A prospective, multicenter, multivendor study. J. Am. Coll. Cardiol..

[14] The SCOT-HEART Investigators (2015). CT coronary angiography in patients with suspected angina due to coronary heart disease (SCOT-HEART): An open-label, parallel-group, multicentre trial. Lancet.

[15] Lampejo T., Durkin S.M., Bhatt N., Guttmann O. (2021). Acute myocarditis: Aetiology, diagnosis and management. Clin. Med..

[16] Sanchez Tijmes F., Thavendiranathan P., Udell J.A., Seidman M.A., Hanneman K. (2021). Cardiac MRI assessment of nonischemic myocardial inflammation: State of the art review and update on myocarditis associated with COVID-19 vaccination. Radiol. Cardiothorac. Imaging.

[17] Fabre A., Sheppard M.N. (2006). Sudden adult death syndrome and other non-ischaemic causes of sudden cardiac death. Heart.

[18] Ammirati E., Veronese G., Bottiroli M., Wang D.W., Cipriani M., Garascia A., Pedrotti P., Adler E.D., Frigerio M. (2021). Update on acute myocarditis. Trends Cardiovasc. Med..

[19] Caforio A.L., Pankuweit S., Arbustini E., Basso C., Gimeno-Blanes J., Felix S.B., Fu M., Helio T., Heymans S., Jahns R. (2013). Current state of knowledge on aetiology, diagnosis, management, and therapy of myocarditis: A position statement of the European society of cardiology working group on myocardial and pericardial diseases. Eur. Heart J..

[20] Tornvall P., Gerbaud E., Behaghel A., Chopard R., Collste O., Laraudogoitia E., Leurent G., Meneveau N., Montaudon M., Perez-David E. (2015). Myocarditis or “true” infarction by cardiac magnetic resonance in patients with a clinical diagnosis of myocardial infarction without obstructive coronary disease: A meta-analysis of individual patient data. Atherosclerosis.

[21] Tschope C., Ammirati E., Bozkurt B., Caforio A.L.P., Cooper L.T., Felix S.B., Hare J.M., Heidecker B., Heymans S., Hubner N. (2021). Myocarditis and inflammatory cardiomyopathy: Current evidence and future directions. Nat. Rev. Cardiol..

[22] Su J.R., McNeil M.M., Welsh K.J., Marquez P.L., Ng C., Yan M., Cano M.V. (2021). Myopericarditis after vaccination, vaccine adverse event reporting system (VAERS), 1990–2018. Vaccine.

[23] Aretz H.T., Billingham M.E., Edwards W.D., Factor S.M., Fallon J.T., Fenoglio J.J., Olsen E.G., Schoen F.J. (1987). Myocarditis. A histopathologic definition and classification. Am. J. Cardiovasc. Pathol..

[24] Lagan J., Fortune C., Hutchings D., Bradley J., Naish J.H., Timoney R., Prescott D., Bain H.D.C., Bangi T., McIntosh J. (2022). The diagnostic and prognostic utility of contemporary cardiac magnetic resonance in suspected acute myocarditis. Diagnostics.

[25] Liguori C., Farina D., Vaccher F., Ferrandino G., Bellini D., Carbone I. (2020). Myocarditis: Imaging up to date. Radiol. Med..

[26] Luetkens J.A., Faron A., Isaak A., Dabir D., Kuetting D., Feisst A., Schmeel F.C., Sprinkart A.M., Thomas D. (2019). Comparison of original and 2018 lake louise criteria for diagnosis of acute myocarditis: Results of a validation cohort. Radiol. Cardiothorac. Imaging.

[27] Lurz P., Eitel I., Adam J., Steiner J., Grothoff M., Desch S., Fuernau G., de Waha S., Sareban M., Luecke C. (2012). Diagnostic performance of CMR imaging compared with EMB in patients with suspected myocarditis. JACC Cardiovasc. Imaging.

[28] Rehwald W.G., Fieno D.S., Chen E.L., Kim R.J., Judd R.M. (2002). Myocardial magnetic resonance imaging contrast agent concentrations after reversible and irreversible ischemic injury. Circulation.

[29] Kalisz K., Halliburton S., Abbara S., Leipsic J.A., Albrecht M.H., Schoepf U.J., Rajiah P. (2017). Update on cardiovascular applications of multienergy CT. Radiographics.

[30] Troughton R.W., Asher C.R., Klein A.L. (2004). Pericarditis. Lancet.

[31] Bogaert J., Francone M. (2013). Pericardial disease: Value of CT and MR imaging. Radiology.

[32] Kligerman S. (2019). Imaging of pericardial disease. Radiol. Clin. N. Am..

[33] Bainey K.R., Welsh R.C., Alemayehu W., Westerhout C.M., Traboulsi D., Anderson T., Brass N., Armstrong P.W., Kaul P. (2018). Population-level incidence and outcomes of myocardial infarction with non-obstructive coronary arteries (MINOCA): Insights from the Alberta contemporary acute coronary syndrome patients invasive treatment strategies (COAPT) study. Int. J. Cardiol..

[34] Safdar B., Spatz E.S., Dreyer R.P., Beltrame J.F., Lichtman J.H., Spertus J.A., Reynolds H.R., Geda M., Bueno H., Dziura J.D. (2018). Presentation, clinical profile, and prognosis of young patients with myocardial infarction with nonobstructive coronary arteries (MINOCA): Results from the VIRGO study. J. Am. Heart Assoc..

[35] Chinali M., Franceschini A., Ciancarella P., Lisignoli V., Curione D., Ciliberti P., Esposito C., Del Pasqua A., Rinelli G., Secinaro A. (2020). Echocardiographic two-dimensional speckle tracking identifies acute regional myocardial edema and sub-acute fibrosis in pediatric focal myocarditis with normal ejection fraction: Comparison with cardiac magnetic resonance. Sci. Rep..

[36] Felker G.M., Boehmer J.P., Hruban R.H., Hutchins G.M., Kasper E.K., Baughman K.L., Hare J.M. (2000). Echocardiographic findings in fulminant and acute myocarditis. J. Am. Coll. Cardiol..

[37] Kindermann I., Kindermann M., Kandolf R., Klingel K., Bultmann B., Muller T., Lindinger A., Bohm M. (2008). Predictors of outcome in patients with suspected myocarditis. Circulation.

[38] Sykes R., Doherty D., Mangion K., Morrow A., Berry C. (2021). What an interventionalist needs to know about MI with non-obstructive coronary arteries. Interv. Cardiol..

[39] Chang S., Han K., Youn J.C., Im D.J., Kim J.Y., Suh Y.J., Hong Y.J., Hur J., Kim Y.J., Choi B.W. (2018). Utility of dual-energy CT-based monochromatic imaging in the assessment of myocardial delayed enhancement in patients with cardiomyopathy. Radiology.

[40] Hanneman K., Kadoch M., Guo H.H., Jamali M., Quon A., Iagaru A., Herfkens R. (2017). Initial experience with simultaneous 18F-FDG PET/MRI in the evaluation of cardiac sarcoidosis and myocarditis. Clin. Nucl. Med..

[41] Pan J.A., Lee Y.J., Salerno M. (2018). Diagnostic performance of extracellular volume, native T1, and T2 mapping versus lake louise criteria by cardiac magnetic resonance for detection of acute myocarditis: A meta-analysis. Circ. Cardiovasc. Imaging.

[42] Ferreira V.M., Schulz-Menger J., Holmvang G., Kramer C.M., Carbone I., Sechtem U., Kindermann I., Gutberlet M., Cooper L.T., Liu P. (2018). Cardiovascular magnetic resonance in nonischemic myocardial inflammation: Expert recommendations. J. Am. Coll. Cardiol..

[43] Satoh H., Sano M., Suwa K., Saitoh T., Nobuhara M., Saotome M., Urushida T., Katoh H., Hayashi H. (2014). Distribution of late gadolinium enhancement in various types of cardiomyopathies: Significance in differential diagnosis, clinical features and prognosis. World J. Cardiol..

[44] Santoro F., Mallardi A., Leopizzi A., Vitale E., Stiermaier T., Trambaiolo P., Di Biase M., Eitel I., Brunetti N.D. (2022). Stepwise approach for diagnosis and management of Takotsubo syndrome with cardiac imaging tools. Heart Fail. Rev..

[45] Assomull R.G., Lyne J.C., Keenan N., Gulati A., Bunce N.H., Davies S.W., Pennell D.J., Prasad S.K. (2007). The role of cardiovascular magnetic resonance in patients presenting with chest pain, raised troponin, and unobstructed coronary arteries. Eur. Heart J..

[46] Citro R., Okura H., Ghadri J.R., Izumi C., Meimoun P., Izumo M., Dawson D., Kaji S., Eitel I., Kagiyama N. (2020). Multimodality imaging in takotsubo syndrome: A joint consensus document of the European association of cardiovascular imaging (EACVI) and the Japanese society of echocardiography (JSE). Eur. Heart J. Cardiovasc. Imaging.

[47] Otsuka Y., Noguchi T., Goto Y., Nonogi H., Yamada N. (2008). Hyperintensity on T2-weighted magnetic resonance imaging in Takotsubo cardiomyopathy. Int. J. Cardiol..

[48] Fernandez-Perez G.C., Aguilar-Arjona J.A., de la Fuente G.T., Samartin M., Ghioldi A., Arias J.C., Sanchez-Gonzalez J. (2010). Takotsubo cardiomyopathy: Assessment with cardiac MRI. AJR Am. J. Roentgenol..

[49] Singh A., Museedi A.S., Grossman S.A. (2022). Acute coronary syndrome. StatPearls.

[50] Ibanez B., James S., Agewall S., Antunes M.J., Bucciarelli-Ducci C., Bueno H., Caforio A.L.P., Crea F., Goudevenos J.A., Halvorsen S. (2018). 2017 ESC Guidelines for the management of acute myocardial infarction in patients presenting with ST-segment elevation: The task force for the management of acute myocardial infarction in patients presenting with ST-segment elevation of the European society of cardiology (ESC). Eur. Heart J..

[51] Lee H.Y., Yoo S.M., White C.S. (2009). Coronary CT angiography in emergency department patients with acute chest pain: Triple rule-out protocol versus dedicated coronary CT angiography. Int. J. Cardiovasc. Imaging.

[52] Yoo S.M., Jang S., Kim J.A., Chun E.J. (2020). Troponin-positive non-obstructive coronary arteries and myocardial infarction with non-obstructive coronary arteries: Definition, etiologies, and role of CT and MR imaging. Korean J. Radiol..

[53] Yoo S.M., Rho J.Y., Lee H.Y., Song I.S., Moon J.Y., White C.S. (2010). Current concepts in cardiac CT angiography for patients with acute chest pain. Korean Circ. J..

[54] Litt H.I., Gatsonis C., Snyder B., Singh H., Miller C.D., Entrikin D.W., Leaming J.M., Gavin L.J., Pacella C.B., Hollander J.E. (2012). CT angiography for safe discharge of patients with possible acute coronary syndromes. N. Engl. J. Med..

[55] Hoffmann U., Truong Q.A., Schoenfeld D.A., Chou E.T., Woodard P.K., Nagurney J.T., Pope J.H., Hauser T.H., White C.S., Weiner S.G. (2012). Coronary CT angiography versus standard evaluation in acute chest pain. N. Engl. J. Med..

[56] Moore A., Goerne H., Rajiah P., Tanabe Y., Saboo S., Abbara S. (2019). Acute myocardial infarct. Radiol. Clin. N. Am..

[57] Kaul U.A., Singh S., Kalra G.S., Nair M., Mohan J.C., Nigam M., Arora R. (2000). Mitral regurgitation following percutaneous transvenous mitral commissurotomy: A single-center experience. J. Heart Valve Dis..

[58] Figueras J., Cortadellas J., Soler-Soler J. (2000). Left ventricular free wall rupture: Clinical presentation and management. Heart.

[59] Son M.J., Yoo S.M., Lee D., Lee H.Y., Song I.S., Chun E.J., White C.S. (2021). Current role of computed tomography in the evaluation of acute coronary syndrome. Diagnostics.

[60] Nardi F., Pino P.G., Gabrielli D., Colivicchi F., Abrignani M.G., Amico A.F., Aspromonte N., Benedetto F.A., Bertella E., Boccardi L.M. (2020). ANMCO/SICI-GISE/SIC/SIECVI/SIRM Consensus document: Appropriateness of multimodality imaging in cardiovascular disease. G. Ital. Cardiol..

[61] Rybicki F.J., Udelson J.E., Peacock W.F., Goldhaber S.Z., Isselbacher E.M., Kazerooni E., Kontos M.C., Litt H., Woodard P.K. (2016). 2015 ACR/ACC/AHA/AATS/ACEP/ASNC/NASCI/SAEM/SCCT/SCMR/SCPC/SNMMI/STR/STS Appropriate utilization of cardiovascular imaging in emergency department patients with chest pain: A joint document of the American college of radiology appropriateness criteria committee and the American college of cardiology appropriate use criteria task force. J. Am. Coll. Cardiol..

[62] Chinnaiyan K.M., Safian R.D., Gallagher M.L., George J., Dixon S.R., Bilolikar A.N., Abbas A.E., Shoukfeh M., Brodsky M., Stewart J. (2020). Clinical use of CT-derived fractional flow reserve in the emergency department. JACC Cardiovasc. Imaging.

[63] Grandhi G.R., Batlle J.C., Maroules C.D., Janowitz W., Peña C.S., Ziffer J.A., Macedo R., Nasir K., Cury R.C. (2021). Combined stress myocardial CT perfusion and coronary CT angiography as a feasible strategy among patients presenting with acute chest pain to the emergency department. J. Cardiovasc. Comput. Tomogr..

[64] Malik N., Claus P.L., Illman J.E., Kligerman S.J., Moynagh M.R., Levin D.L., Woodrum D.A., Arani A., Arunachalam S.P., Araoz P.A. (2017). Air embolism: Diagnosis and management. Future Cardiol..

[65] Shamshirsaz A.A., Clark S.L. (2016). Amniotic fluid embolism. Obstet. Gynecol. Clin. N. Am..

[66] Focardi M., Bonelli A., Pinchi V., De Luca F., Norelli G.A. (2016). Pulmonary cement embolism after kyphoplasty. J. Forensic Sci..

[67] Fedullo P.F., Auger W.R., Kerr K.M., Rubin L.J. (2001). Chronic thromboembolic pulmonary hypertension. N. Engl. J. Med..

[68] Cohen A.T., Agnelli G., Anderson F.A., Arcelus J.I., Bergqvist D., Brecht J.G., Greer I.A., Heit J.A., Hutchinson J.L., Kakkar A.K. (2007). Venous thromboembolism (VTE) in Europe. The number of VTE events and associated morbidity and mortality. Thromb. Haemost..

[69] Konstantinides S. (2020). 2019 ESC guidelines on pulmonary embolism: Novelties and unanswered questions. Eur. J. Intern. Med..

[70] Zantonelli G., Cozzi D., Bindi A., Cavigli E., Moroni C., Luvara S., Grazzini G., Danti G., Granata V., Miele V. (2022). Acute pulmonary embolism: Prognostic role of computed tomography pulmonary angiography (CTPA). Tomography.

[71] Kearon C., de Wit K., Parpia S., Schulman S., Afilalo M., Hirsch A., Spencer F.A., Sharma S., D’Aragon F., Deshaies J.F. (2019). Diagnosis of pulmonary embolism with d-dimer adjusted to clinical probability. N. Engl. J. Med..

[72] Tanabe Y., Landeras L., Ghandour A., Partovi S., Rajiah P. (2018). State-of-the-art pulmonary arterial imaging—Part 1. Vasa.

[73] Qanadli S.D., El Hajjam M., Vieillard-Baron A., Joseph T., Mesurolle B., Oliva V.L., Barre O., Bruckert F., Dubourg O., Lacombe P. (2001). New CT index to quantify arterial obstruction in pulmonary embolism: Comparison with angiographic index and echocardiography. AJR Am. J. Roentgenol..

[74] Sista A.K., Kuo W.T., Schiebler M., Madoff D.C. (2017). Stratification, imaging, and management of acute massive and submassive pulmonary embolism. Radiology.

[75] Rotzinger D.C., Knebel J.F., Jouannic A.M., Adler G., Qanadli S.D. (2020). CT Pulmonary angiography for risk stratification of patients with nonmassive acute pulmonary embolism. Radiol. Cardiothorac. Imaging.

[76] Cho S.U., Cho Y.D., Choi S.H., Yoon Y.H., Park J.H., Park S.J., Lee E.S. (2020). Assessing the severity of pulmonary embolism among patients in the emergency department: Utility of RV/LV diameter ratio. PLoS ONE.

[77] Apfaltrer P., Henzler T., Meyer M., Roeger S., Haghi D., Gruettner J., Suselbeck T., Wilson R.B., Schoepf U.J., Schoenberg S.O. (2012). Correlation of CT angiographic pulmonary artery obstruction scores with right ventricular dysfunction and clinical outcome in patients with acute pulmonary embolism. Eur. J. Radiol..

[78] Ayoz S., Erol S., Kul M., Gurun Kaya A., Gursoy Coruh A., Savas I., Aydin O., Kaya A. (2021). Using RV/LV ratio and cardiac biomarkers to define the risk of mortality from pulmonary embolism. Tuberk Toraks.

[79] Cozzi D., Moroni C., Cavigli E., Bindi A., Caviglioli C., Nazerian P., Vanni S., Miele V., Bartolucci M. (2021). Prognostic value of CT pulmonary angiography parameters in acute pulmonary embolism. Radiol. Med..

[80] Akhoundi N., Langroudi T.F., Rajebi H., Haghi S., Paraham M., Karami S., Langroudi F.K. (2019). Computed tomography pulmonary angiography for acute pulmonary embolism: Prediction of adverse outcomes and 90-day mortality in a single test. Pol. J. Radiol..

[81] Aviram G., Soikher E., Bendet A., Shmueli H., Ziv-Baran T., Amitai Y., Friedensohn L., Berliner S., Meilik A., Topilsky Y. (2016). Prediction of mortality in pulmonary embolism based on left atrial volume measured on CT pulmonary angiography. Chest.

[82] Williams M.C., Morley N.C.D., Muir K.C., Reid J.H., van Beek E.J.R., Murchison J.T. (2019). Coronary artery calcification is associated with mortality independent of pulmonary embolism severity: A retrospective cohort study. Clin. Radiol..

[83] Palm V., Rengier F., Rajiah P., Heussel C.P., Partovi S. (2020). Acute pulmonary embolism: Imaging techniques, findings, endovascular treatment and differential diagnoses. Rofo.

[84] Norton L., Cooper G., Sheerins O., Mac A’ Bhaird K., Roditi G., Adamson M., Young D., Dolan R., Church C., Brady A. (2021). Clinical and radiological characteristics of acute pulmonary embolus in relation to 28-day and 6-month mortality. PLoS ONE.

[85] Vedovati M.C., Germini F., Agnelli G., Becattini C. (2013). Prognostic role of embolic burden assessed at computed tomography angiography in patients with acute pulmonary embolism: Systematic review and meta-analysis. J. Thromb. Haemost..

[86] Weekes A.J., Raper J.D., Lupez K., Thomas A.M., Cox C.A., Esener D., Boyd J.S., Nomura J.T., Davison J., Ockerse P.M. (2021). Development and validation of a prognostic tool: Pulmonary embolism short-term clinical outcomes risk estimation (PE-SCORE). PLoS ONE.

[87] Lyhne M.D., Schultz J.G., MacMahon P.J., Haddad F., Kalra M., Tso D.M., Muzikansky A., Lev M.H., Kabrhel C. (2019). Septal bowing and pulmonary artery diameter on computed tomography pulmonary angiography are associated with short-term outcomes in patients with acute pulmonary embolism. Emerg. Radiol..

[88] Moore A.J.E., Wachsmann J., Chamarthy M.R., Panjikaran L., Tanabe Y., Rajiah P. (2018). Imaging of acute pulmonary embolism: An update. Cardiovasc. Diagn. Ther..

[89] Reinartz P., Wildberger J.E., Schaefer W., Nowak B., Mahnken A.H., Buell U. (2004). Tomographic imaging in the diagnosis of pulmonary embolism: A comparison between V/Q lung scintigraphy in SPECT technique and multislice spiral CT. J. Nucl. Med..

